# Genetic relationship between *IL-10* gene polymorphisms and the risk of clinical atopic dermatitis

**DOI:** 10.1186/s12881-019-0817-8

**Published:** 2019-05-17

**Authors:** Yuqing Qi, Jie Kong, Jinyan He

**Affiliations:** 1 Department of Dermatology and Venereology, Tianjin Medical University General Hospital, Tianjin Medical University, Tianjin, 300052 People’s Republic of China; 2 Department of Physiology and Pathophysiology, School of Basic Medical Sciences, Tianjin Medical University, No. 22 Qixiangtai Road, Tianjin, 300070 People’s Republic of China

**Keywords:** *IL-10*, Atopic dermatitis, Polymorphism, Susceptibility

## Abstract

**Background:**

We retrieved different reports containing different genetic effects of − 1082 A/G, − 819 T/C, and − 592 A/C polymorphisms within the *IL-10 (interleukin-10)* gene on the susceptibility to clinical atopic dermatitis.

**Methods:**

Herein, we conducted a meta-analysis to comprehensively assess such a genetic relationship after collecting the available published evidence. STATA 12.0 software was used for the statistical analysis under the allelic, homozygotic, heterozygotic, dominant, recessive and carrier genetic models.

**Results:**

By retrieving and screening database literature, a total of 16 eligible case-control studies were finally selected. For the *IL-10* -1082 A/G polymorphism, we did not detect a significant difference between atopic dermatitis cases and population-based controls in the overall meta-analysis under the genetic models of allele G vs. A (*P* = 0.540), GG vs. AA (*P* = 0.853), AG vs AA (*P* = 0.265), AG + GG vs AA (*P* = 0.221), GG vs AA+AG (*P* = 0.540) and carrier G vs. A (*P* = 0.643). Moreover, a statistically non-significant association was observed in the most subgroup meta-analyses by the factors of ethnicity, country and Hardy-Weinberg equilibrium. Likewise, the negative results were detected for the synthetic analysis of *IL-10* -819 T/C and − 592 C/A polymorphisms.

**Conclusion:**

The current evidence does not support a strong genetic relationship between *IL-10* -1082 A/G, − 819 T/C and − 592 A/C polymorphisms and the susceptibility to atopic dermatitis.

**Electronic supplementary material:**

The online version of this article (10.1186/s12881-019-0817-8) contains supplementary material, which is available to authorized users.

## Background

Clinical atopic dermatitis is a common and chronic inflammatory disorder of the epithelial barrier with relapsing eczematous lesions and intense irritation [1–3]. The pathogenesis of atopic dermatitis remains unclear. The skin microbiome, genetics and innate/adaptive immune responses are closely related to the incidence and development of atopic dermatitis [4–9]. For example, a genome-wide association study (GWAS) of 246 recalcitrant atopic dermatitis patients and 551 negative controls from the Korean population reported susceptibility loci within the 13q21.31 region [9]. In the current study, we are interested in quantitatively investigating the possible effect of *interleukin-10* (*IL-10*) polymorphisms on susceptibility to atopic dermatitis.

The IL-10 cytokine participates in the modulation of acquired immune and anti-inflammatory responses [10, 11]. Within the promoter region of the *IL-10* gene, three common single nucleotide polymorphisms (SNPs), namely, − 1082 A/G (rs1800896), − 819 T/C (rs1800871) and − 592 A/C (rs1800872), were identified [12–14]. Different results regarding the genetic effect of *IL-10* polymorphisms on the risk of atopic dermatitis were reported by individual researchers. For example, the *IL-10* -1082 A/G polymorphism was reportedly linked to susceptibility to atopic dermatitis in patients from India [15], Italy [16], and the Czech Republic [17]. Nevertheless, the *IL-10* -1082 A/G polymorphism was not associated with atopic dermatitis risk in the Korean population [18] or Polish population [19]. In addition, the TT genotype frequency of the *IL-10* -819 T/C polymorphism in atopic dermatitis patients was higher than that in healthy control subjects in India [15]. However, the CC genotype of the *IL-10* -819 T/C polymorphism was also reported to be associated with an enhanced risk of severe atopic dermatitis in patients from the Czech Republic [17]. A negative association between the *IL-10* -819 T/C polymorphism and atopic dermatitis risk was also reported in Saudi Arabia [20] and Taiwan of China [21]. This issue merits the performance of a relative meta-analysis.

Some meta-analyses examined the variable degrees of the effect of *IL-10* SNPs on several skin-associated clinical diseases. For instance, the *IL-10* -819 T/C polymorphism, rather than − 1082 A/G and − 592 A/C, was reported to be associated with a decreased risk of skin cancer [22]. In 2013, Chen, et al. conducted a meta-analysis to assess the possible impact of the *IL-10* -1082 A/G polymorphism on the risk of atopic dermatitis [23]. Along with the enrolment of new publications, it is important to conduct an updated meta-analysis to reassess the role of the *IL-10* polymorphisms in the risk of atopic dermatitis.

## Methods

### Database retrieval

**Fig. 1:**
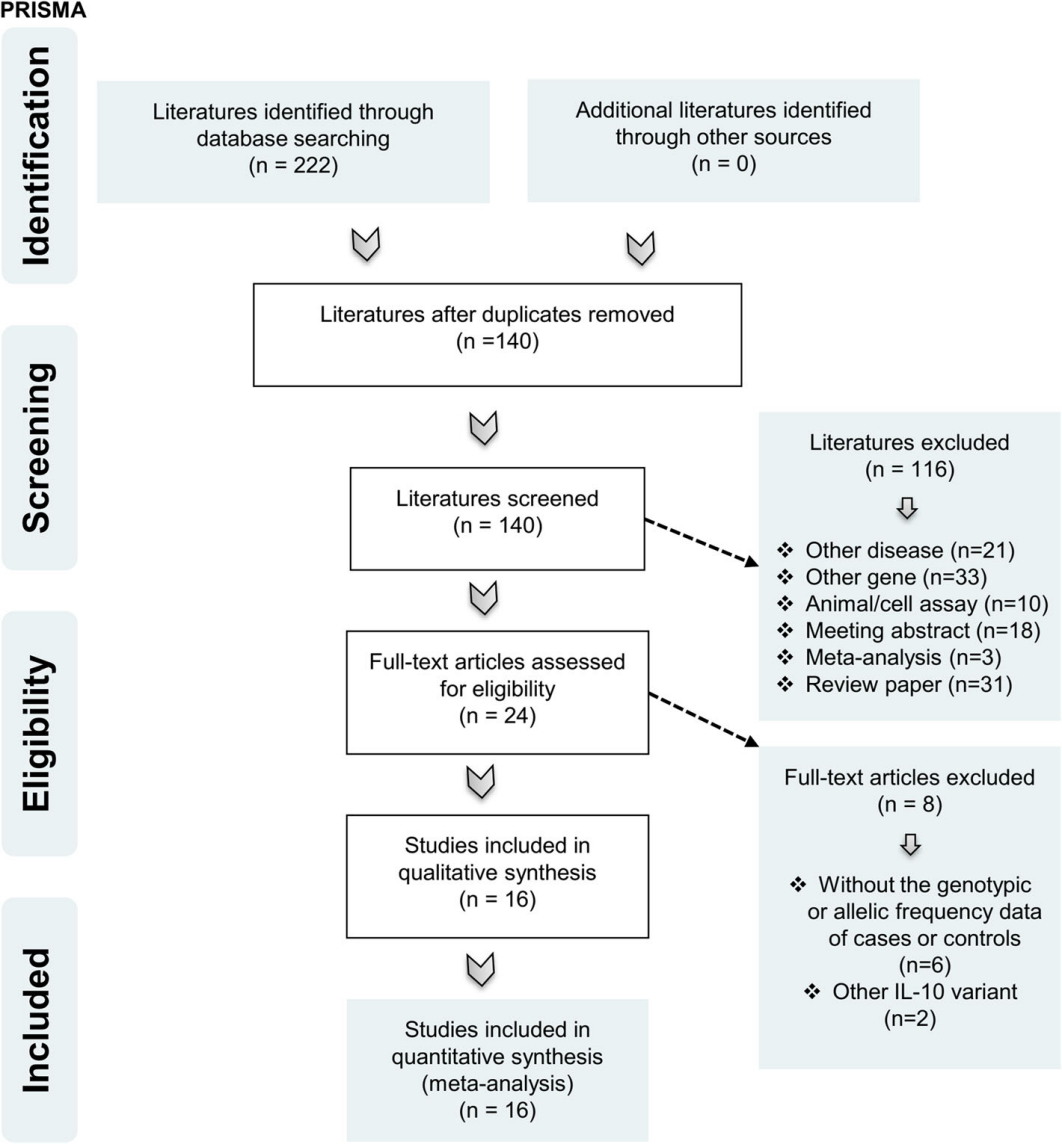
PRISMA-based flow chart for study enrolment

Our meta-analysis follows the guide of preferred reporting items for systematic reviews and meta-analyses (PRISMA) [24]. The PRISMA-based flow chart is shown in Fig. 1. In April 2019, five online electronic databases, including PubMed, Embase, Web of Science (WOS), China National Knowledge Infrastructure (CNKI) and WANFANG, were used to retrieve data by combining the different terms of “IL-10”, “atopic dermatitis” and “polymorphism”. The search strategy for PubMed containing the Medical Subject Headings (MeSH) and entry terms was as follows: ((((((((Interleukin-10 [MeSH Terms]) OR Interleukin 10) OR IL10) OR IL-10) OR CSIF-10) OR Cytokine Synthesis Inhibitory Factor)) AND (((((((((((((((((Dermatitis, Atopic [MeSH Terms]) OR Atopic Dermatitides) OR Atopic Dermatitis) OR Dermatitides, Atopic) OR Neurodermatitis, Atopic) OR Atopic Neurodermatitides) OR Atopic Neurodermatitis) OR Neurodermatitides, Atopic) OR Neurodermatitis, Disseminated) OR Disseminated Neurodermatitides) OR Disseminated Neurodermatitis) OR Neurodermatitides, Disseminated) OR Eczema, Atopic) OR Atopic Eczema) OR Eczema, Infantile) OR Infantile Eczema))) AND ((((((Polymorphism, Genetic [MeSH Terms]) OR Polymorphisms, Genetic) OR Genetic Polymorphisms) OR Genetic Polymorphism) OR Polymorphism (Genetics)) OR Polymorphisms (Genetics)). The search strategy for Embase containing the Emtree and Synonyms: (‘atopic dermatitis’/exp. OR ‘atopic eczema’ OR ‘coca sulzberger disease’ OR ‘coca sulzberger syndrome’ OR ‘dermatitis, atopic’ OR ‘eczema atopica’ OR ‘eczema endogenous’ OR ‘eczema infantum’ OR ‘eczema, infantile’ OR ‘endogenous eczema’ OR ‘infantile eczema’ OR ‘neurodermatitis constitutionalis’ OR ‘neurodermatitis disseminata’ OR ‘neurodermatitis, atopic constitutional’) AND (‘il10’ OR ‘csif’ OR ‘cytokine synthesis inhibitory factor’ OR ‘il 10’ OR ‘il-10’ OR ‘interleukin-10’) AND (‘genetic polymorphism’/exp. OR ‘polymorphism (genetics)’ OR ‘polymorphism, genetic’).

### Study selection

After database retrieval, we designed our inclusion and exclusion criteria to select the eligible case-control studies for synthetic analysis. Exclusion criteria: (a) other diseases; (b) other genes; (c) animal/cell assay; (d) meeting abstract; (e) meta-analysis; (f) review paper; (g) other IL-10 variant; and (h) duplicate study. Inclusion criteria: (a) case and control study; (b) atopic dermatitis; (c) -1082 A/G, − 819 T/C, − 592 A/C polymorphisms within the *IL-10* gene; and (d) the allelic or genotypic frequency data of cases and controls.

### Data extraction

Subsequently, we extracted basic information, including first author, publication time (year), ethnicity, SNPs, genotypic or allelic frequency data in both controls and cases, control source, genotyping method, sample size, and *P* value of Hardy-Weinberg equilibrium (HWE). The Newcastle-Ottawa quality assessment Scale (NOS) scores of study quality were also measured.

### Statistical analysis

After referring to several reported meta-analyses [25–27], we utilized STATA 12.0 software (Stata Corporation, College Station, TX, USA) to conduct the I^2^ test and Q statistic test (for heterogeneity evaluation), DerSimonian-Laird and Mantel-Haenszel method (for association test), Begg’s test and Egger’s test (for publication bias evaluation) [28, 29], and sensitivity analysis (for the assessment of data stability). The high heterogeneity level was considered to perform the DerSimonian-Laird method under a random-effect model when I^2^ was larger than 50% or the *P* value was less than 0.05. In contrast, the Mantel-Haenszel method under a fixed-effect model was used for the association test. In addition, the data of the odds ratio (OR), 95% confidence interval (CI) and *P* value were calculated under the allelic, homozygotic, heterozygotic, dominant, recessive and carrier genetic models in the overall meta-analysis as well as the relative subgroup analysis by the factors of ethnicity, country and HWE. Only the pooling results from at least three case-control studies were considered in our study.

## Results

### Enrolled studies

As shown in Fig. 1, we initially identified a total of 222 studies, including PubMed (*n* = 28), Embase (*n* = 79), WOS (*n* = 104), CNKI (n = 1) and WANFANG (*n* = 10), and we removed the 82 duplicates. Additionally, we ruled out another 116 studies by the exclusion criteria shown in Fig. 1. Moreover, after the eligibility assessment, we eliminated another six articles without the genotypic or allelic frequency data in cases or controls, and two articles regarding the other *IL-10* variant were excluded. As a result, a total of 16 eligible case-control studies [15–21, 30–38] with the summarized basic information in Table 1 were enrolled. We observed population-based controls and high-quality controls (Additional file 1: Table S1, all NOS scores greater than five) in each study.

**Table 1:** Basic information for the studies included in the meta-analysis

First author, Year	[Ref.]	Ethnicity	Country	SNPs	Control		Case	Genotyping method	HWE P
					XX/XY/YY	Source	XX/XY/YY		
Arkwright, 2001	[32]	Mixed	UK	-1082A/G	16/21/13	PB	22/30/16	ARMS-PCR	0.27
Babic, 2016	[31]	Caucasian	Croatia	-1082A/G	25/107*	PB	9/28*	KASP + competitive ASPCR	>0.05
Behniafard, 2018	[37]	Asian	Iran	-1082A/G	53/75/12	PB	30/46/13	PCR-SSP	0.04
				-819 T/C	12/57/71	PB	6/42/41	PCR-SSP	0.91
				-592A/C	12/57/71	PB	5/42/42	PCR-SSP	0.91
Bin, 2018	[20]	Asian	Saudi Arabia	-1082A/G	36/159/16	PB	30/53/21	ARMS-PCR	< 0.05
				-819 T/C	21/102/88	PB	14/54/36	ARMS-PCR	0.27
				-592A/C	21/102/88	PB	14/54/36	ARMS-PCR	0.27
Chang, 2006	[21]	Asian	China	-1082A/G	344^#^/28^#^	PB	174^#^/14^#^	gene sequecing	>0.05
				-819 T/C	250^#^/122^#^	PB	132^#^/56^#^	gene sequecing	>0.05
				-592A/C	250^#^/122^#^	PB	132^#^/56^#^	gene sequecing	>0.05
Esposito, 2015	[16]	Caucasian	Italy	-1082A/G	38/60/20	PB	50/41/10	TaqMan Array	0.65
				-592A/C	6/39/73	PB	13/49/42	TaqMan Array	0.79
Jain, 2017	[15]	Asian	India	-1082A/G	7/31/0	PB	23/15/0	ARMS-PCR	< 0.05
				-819 T/C	1/28/9	PB	8/26/4	ARMS-PCR	< 0.05
				-592A/C	1/28/9	PB	8/26/4	ARMS-PCR	< 0.05
Kayserova, 2012	[17]	Caucasian	Czech Republic	-1082A/G	20/71/11	PB	29/43/16	PCR-SSP	< 0.05
				-819 T/C	4/50/48	PB	7/26/55	PCR-SSP	0.04
				-592A/C	5/48/49	PB	8/25/55	PCR-SSP	0.11
Lesiak, 2011	[19]	Caucasian	Poland	-1082A/G	67/89/48	PB	54/75/34	PCR-RFLP	0.09
Lesiak, 2014	[33]	Caucasian	Poland	-1082A/G	20/26/14	PB	25/35/16	PCR-RFLP	0.33
Reich, 2003	[34]	Caucasian	Germany	-1082A/G	49/118/47	PB	18/53/23	PCR-RFLP/sequencing	0.13
Sheng, 2018	[38]	Asian	China	-819 T/C	128/130/41	PB	83/154/63	Sequenom MassARRAY	0.39
Sohn, 2007	[18]	Asian	Korea	-1082A/G	124/15/1	PB	241/35/0	PCR-RFLP/sequencing	0.47
				-819 T/C	66/68/6	PB	145/104/27	PCR-RFLP/sequencing	0.02
				-592A/C	64/65/11	PB	137/110/29	PCR-RFLP/sequencing	0.32
Stavric, 2012	[35]	Caucasian	Macedonia	-1082A/G	70/212/17	PB	8/55/3	PCR-SSP	< 0.05
				-819 T/C	155/125/19	PB	39/27/0	PCR-SSP	0.35
				-592A/C	28/117/154	PB	3/27/36	PCR-SSP	0.40
Yinji, 2010	[30]	Asian	China	-1082A/G	109/20/0	PB	143/30/0	TaqMan	0.34
				-819 T/C	55/60/14	PB	89/70/14	TaqMan	0.69
				-592A/C	55/60/14	PB	89/70/14	TaqMan	0.69
Zakrzewski, 2010	[36]	Caucasian	Poland	-1082A/G	67/89/48	PB	54/75/34	PCR-RFLP	0.09

*Ref.* reference, SNPs single nucleotide polymorphisms, *XX* AA genotype (−1082 A/G SNP), TT genotype (−819 T/C SNP), AA genotype (−592 A/C SNP), *XY* AG genotype (−1082 A/G SNP), TC genotype (−819 T/C SNP), AC genotype (−592 A/C SNP), *YY* GG genotype (− 1082 A/G SNP), CC genotype (− 819 T/C SNP), CC genotype (− 592 A/C SNP), *PB* population-based control, *ARMS* amplification refractory mutational system, *PCR* polymerase chain reaction; *KASP* Kompetitive Allele Specific PCR, *ASPCR* allele-specific PCR, *SSP* sequence specific primer, *RFLP* restriction fragment-length polymorphism, *HWE* Hardy-Weinberg equilibrium

* the frequency data of AG + GG; # the frequency data of X, Y allele

### Meta-analysis of *IL-10* -1082 a/G SNP

**Fig. 2:**
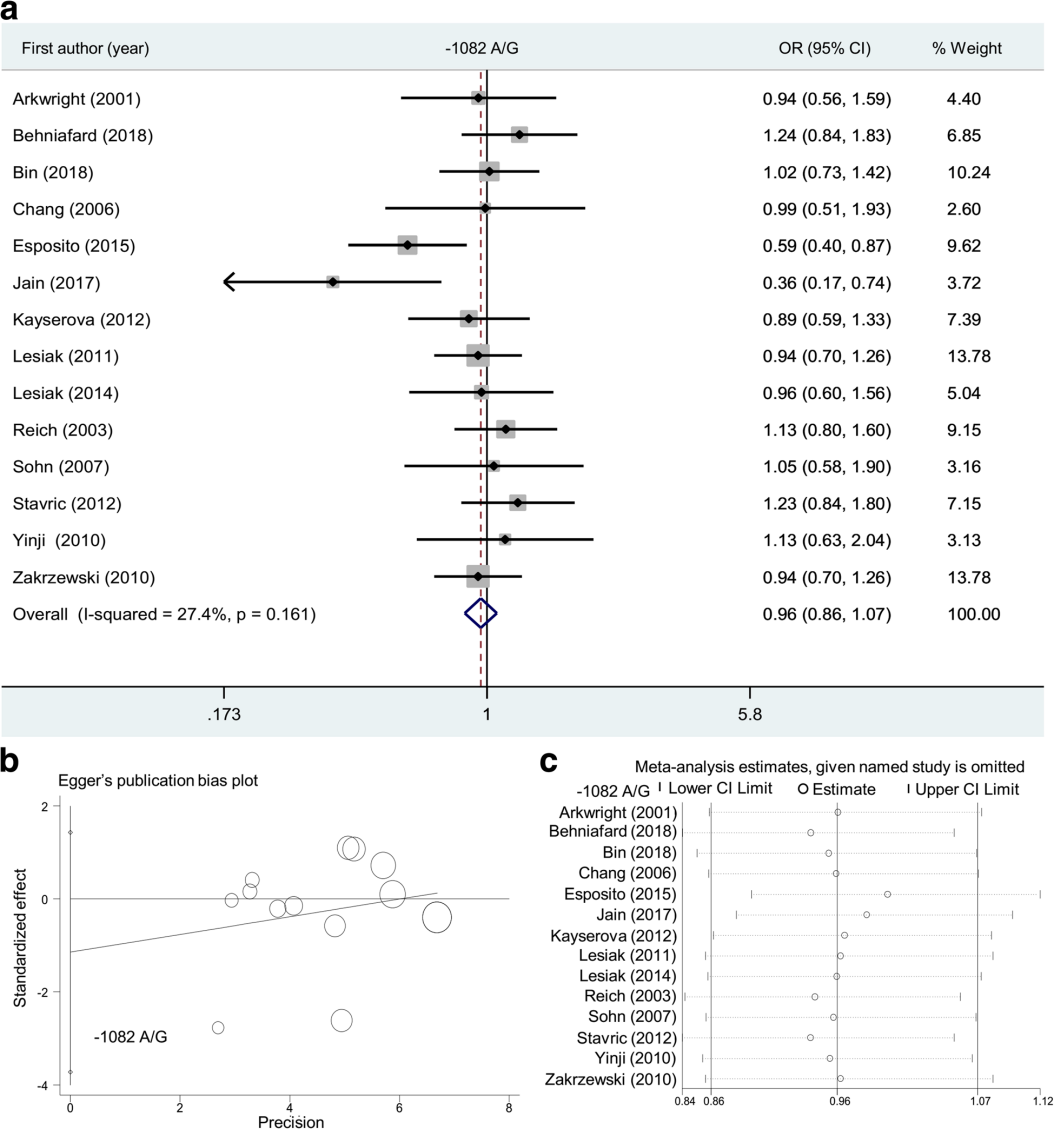
Overall meta-analysis of the *IL-10* -1082 A/G SNP under the allelic model. **a** Forest plot; (**b**) Egger’s publication bias plot; (**c**) Sensitivity analysis

During the meta-analysis of the *IL-10* -1082 A/G polymorphism (Table 2), 14 case-control studies were enrolled for the models of allele G vs. A (1,593 cases/2095 controls) and AG + GG vs. AA (1536 cases/2041 controls); 13 studies (1499 cases/1909 control) for the AG vs AA and carrier G vs. A; 11 studies (1288 cases/1742 controls) for the models of GG vs. AA and GG vs. AA+AG. In the association test shown in Table 2, we did not detect a significant difference between atopic dermatitis cases and population-based controls in the overall meta-analysis under the genetic models of allele G vs. A (*P* = 0.540), GG vs. AA (*P* = 0.853), AG vs AA (*P* = 0.265), AG + GG vs AA (*P* = 0.221), GG vs AA+AG (*P* = 0.540) and carrier G vs. A (*P* = 0.643). Likewise, we observed a statistically non-significant association in the subgroup analysis of “Asian”, “Caucasian”, “Poland”, and “Y, with a *P* value of HWE >0.05” (Table 2, *P* > 0.05), only apart from the “Asian” subgroup under the GG vs AA+AG model (*P* = 0.003, OR = 2.22). However, when we excluded the studies with the *P* value of HWE < 0.05, the negative results were obtained in the subgroup analysis of “Asian/Y”, “Caucasian/Y” and “Poland/Y” (Table 2, all *P* > 0.05). Forest plot data of overall meta-analysis under the allelic model (Fig. 2a) and subgroup analysis by ethnicity under allelic (Additional file 2: Figure S1), heterozygotic (Additional file 2: Figure S2) and dominant (Additional file 2: Figure S3) models were provided. In summary, the *IL-10* -1082 A/G SNP may have no genetic effect on the risk of atopic dermatitis.

**Table 2:** Meta-analysis of *IL-10* -1082 A/G SNP

Genetic model	Group	Study number	Case/Control	*P*	OR (95% CI)
Allele G vs. A	Overall	14	1593/2095	0.540	0.96 (0.86~1.07)
	Asian	6	774/844	0.990	1.00 (0.82~1.22)
	Caucasian	7	751/1201	0.395	0.94 (0.83~1.08)
	Poland	3	402/468	0.562	0.95 (0.78~1.14)
	Y	9	1208/1305	0.326	0.93 (0.82~1.07)
	Asian/Y	3	543/455	0.751	1.06 (0.74~1.51)
	Caucasian/Y	5	597/800	0.238	0.91 (0.78~1.06)
	Poland/Y	3	402/468	0.562	0.95 (0.78~1.14)
GG vs. AA	Overall	11	1288/1742	0.853	0.98 (0.76~1.25)
	Asian	3	469/491	0.144	1.55 (0.86~2.78)
	Caucasian	7	751/1201	0.390	0.88 (0.66~1.17)
	Poland	3	402/468	0.513	0.88 (0.61~1.28)
	Y	7	941/990	0.241	0.84 (0.63~1.12)
	Caucasian/Y	5	597/800	0.299	0.85 (0.63~1.15)
	Poland/Y	3	402/468	0.513	0.88 (0.61~1.28)
AG vs AA	Overall	13	1499/1909	0.265	0.84 (0.62~1.14)
	Asian	5	680/658	0.216	0.66 (0.35~1.27)
	Caucasian	7	751/1201	0.720	0.93 (0.65~1.35)
	Poland	3	402/468	0.753	1.05 (0.77~1.43)
	Y	8	1114/1119	0.975	1.00 (0.81~1.23)
	Caucasian/Y	5	597/800	0.683	0.94 (0.71~1.26)
	Poland/Y	3	402/468	0.753	1.05 (0.77~1.43)
AG + GG vs AA	Overall	14	1536/2041	0.221	0.85 (0.65~1.10)
	Asian	5	680/658	0.268	0.71 (0.39~1.30)
	Caucasian	8	788/1333	0.502	0.90 (0.66~1.23)
	Poland	3	402/468	0.957	0.99 (0.75~1.32)
	Y	9	1151/1251	0.521	0.94 (0.77~1.14)
	Caucasian/Y	6	634/932	0.369	0.88 (0.68~1.16)
	Poland/Y	3	402/468	0.957	0.99 (0.75~1.32)
GG vs AA + AG	Overall	11	1288/1742	0.540	1.07 (0.86~1.32)
	Asian	3	469/491	0.003	2.22 (1.32~3.74)
	Caucasian	7	751/1201	0.538	0.93 (0.72~1.18)
	Poland	3	402/468	0.358	0.86 (0.62~1.19)
	Y	7	941/990	0.233	0.86 (0.67~1.10)
	Caucasian/Y	5	597/800	0.295	0.87 (0.67~1.13)
	Poland/Y	3	402/468	0.358	0.86 (0.62~1.19)
Carrier G vs. A	Overall	13	1499/1909	0.643	0.97 (0.86~1.10)
	Asian	5	680/658	0.968	1.00 (0.79~1.25)
	Caucasian	7	751/1201	0.608	0.96 (0.82~1.12)
	Poland	3	402/468	0.751	0.96 (0.77~1.21)
	Y	8	1114/1119	0.619	0.96 (0.82~1.13)
	Caucasian/Y	5	597/800	0.475	0.94 (0.78~1.12)
	Poland/Y	3	402/468	0.751	0.96 (0.77~1.21)

*OR* odds ratio, *Y P* value of HWE >0.05, *CI* confidence interval

### Meta-analysis of *IL-10* -819 T/C SNP

**Fig. 3:**
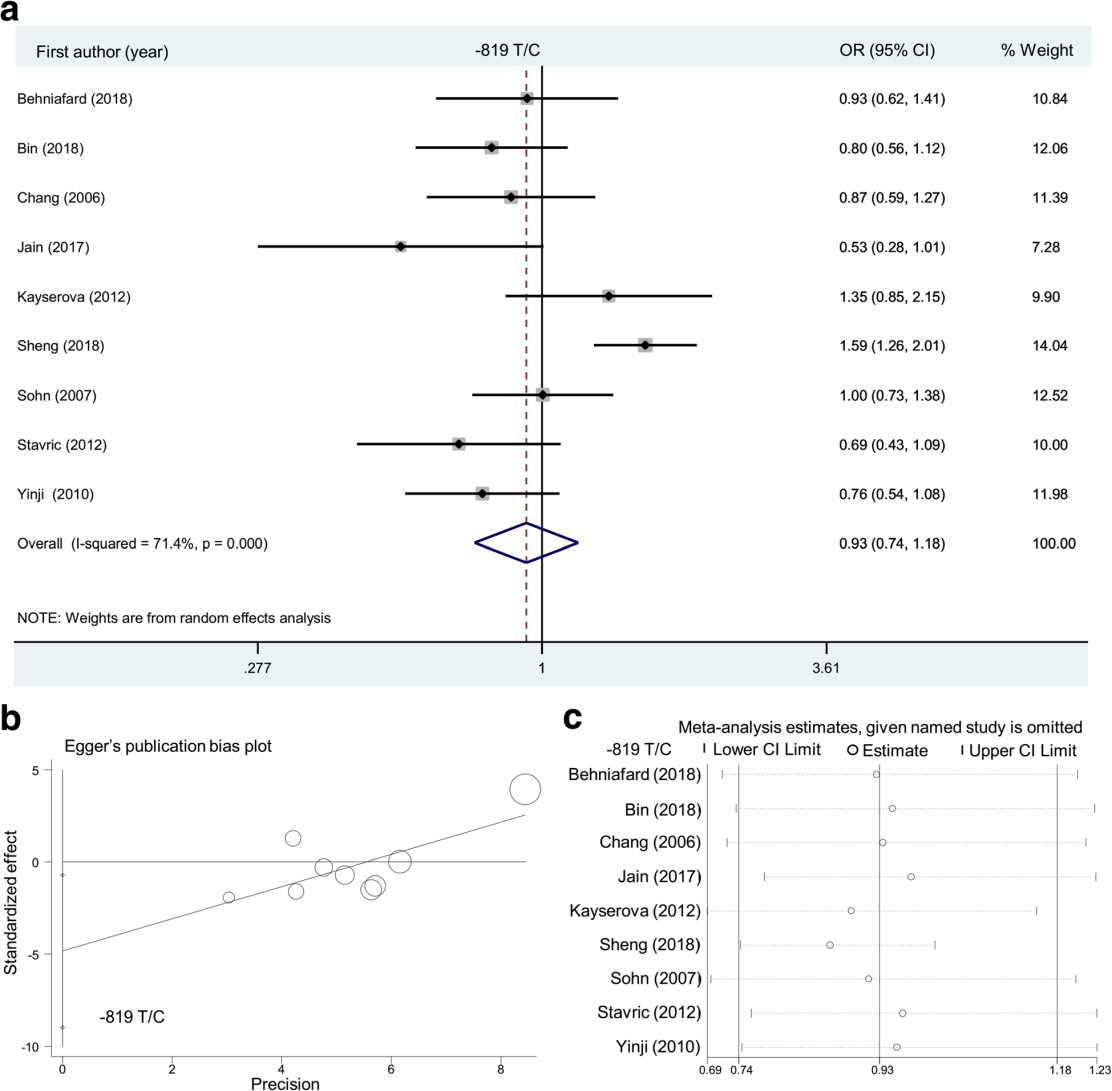
Overall meta-analysis of the *IL-10* -819 T/C SNP under the allelic model. (**a**) Forest plot; (**b**) Egger’s publication bias plot; (**c**) Sensitivity analysis

In addition, for the *IL-10* -819 T/C SNP (Table 3), nine case-control studies (1228/1544) were included in the allelic model, while eight studies (1134/1358) were included in other models. As shown in Table 2, negative results were detected in cases compared with that in population-based controls during the overall meta-analysis under the models of allele C vs. T (*P* = 0.547), CC vs. TT (*P* = 0.588), TC vs. TT (*P* = 0.405), TC + CC vs. TT (*P* = 0.433), CC vs. TT + TC (*P* = 0.821), and carrier C vs. T (*P* = 0.801). Likewise, we observed a non-significant association in the subgroup analysis of “Asian”, “Y” and “Asian/Y”, under all genetic models (Table 3, all *P* > 0.05). The relative forest plots are displayed in Fig. 3a (overall meta-analysis under the allelic model), Additional file 2: Figure S4 (subgroup analysis by ethnicity under the allelic model), Additional file 2: Figure S5 (subgroup analysis by ethnicity under the heterozygotic model), and Additional file 2: Figure S6 (subgroup analysis by ethnicity under the dominant model). Our findings suggested that the *IL-10* -819 T/C SNP may not be associated with the risk of atopic dermatitis.

**Table 3:** Meta-analysis of *IL-10* -819 T/C SNP

Genetic model	Group	Study number	Case/Control	*P*	OR (95% CI)
Allele C vs. T	Overall	9	1228/1544	0.547	0.93 (0.74~1.18)
	Asian	7	1074/1143	0.550	0.92 (0.71~1.20)
	China	3	567/614	0.893	1.03 (0.63~1.70)
	Y	7	1102/1404	0.610	0.94 (0.73~1.21)
	Asian/Y	6	1036/1105	0.866	0.98 (0.75~1.28)
CC vs. TT	Overall	8	1134/1358	0.588	0.83 (0.42~1.63)
	Asian	6	980/957	0.915	0.96 (0.46~1.99)
	Y	6	1008/1218	0.870	1.06 (0.54~2.07)
	Asian/Y	5	942/919	0.593	1.19 (0.63~2.26)
TC vs. TT	Overall	8	1134/1358	0.405	0.83 (0.55~1.28)
	Asian	6	980/957	0.697	0.90 (0.54~1.51)
	Y	6	1008/1218	0.892	0.97 (0.65~1.45)
	Asian/Y	5	942/919	0.995	1.00 (0.62~1.62)
TC + CC vs. TT	Overall	8	1134/1358	0.433	0.84 (0.55~1.30)
	Asian	6	980/957	0.693	0.90 (0.53~1.52)
	Y	6	1008/1218	0.845	0.96 (0.63~1.47)
	Asian/Y	5	942/919	0.975	1.01 (0.61~1.66)
CC vs. TT + TC	Overall	8	1134/1358	0.821	1.05 (0.69~1.60)
	Asian	6	980/957	0.991	1.00 (0.64~1.55)
	Y	6	1008/1218	0.900	1.03 (0.65~1.64)
	Asian/Y	5	942/919	0.706	1.09 (0.70~1.70)
Carrier C vs. T	Overall	8	1134/1358	0.801	1.02 (0.88~1.17)
	Asian	6	980/957	0.816	1.02 (0.87~1.19)
	Y	6	1008/1218	0.867	1.01 (0.87~1.18)
	Asian/Y	5	942/919	0.631	1.04 (0.89~1.22)

*OR* odds ratio, *Y P* value of HWE >0.05, *CI* confidence interval

### Meta-analysis of *IL-10* -592 a/C SNP

**Fig. 4:**
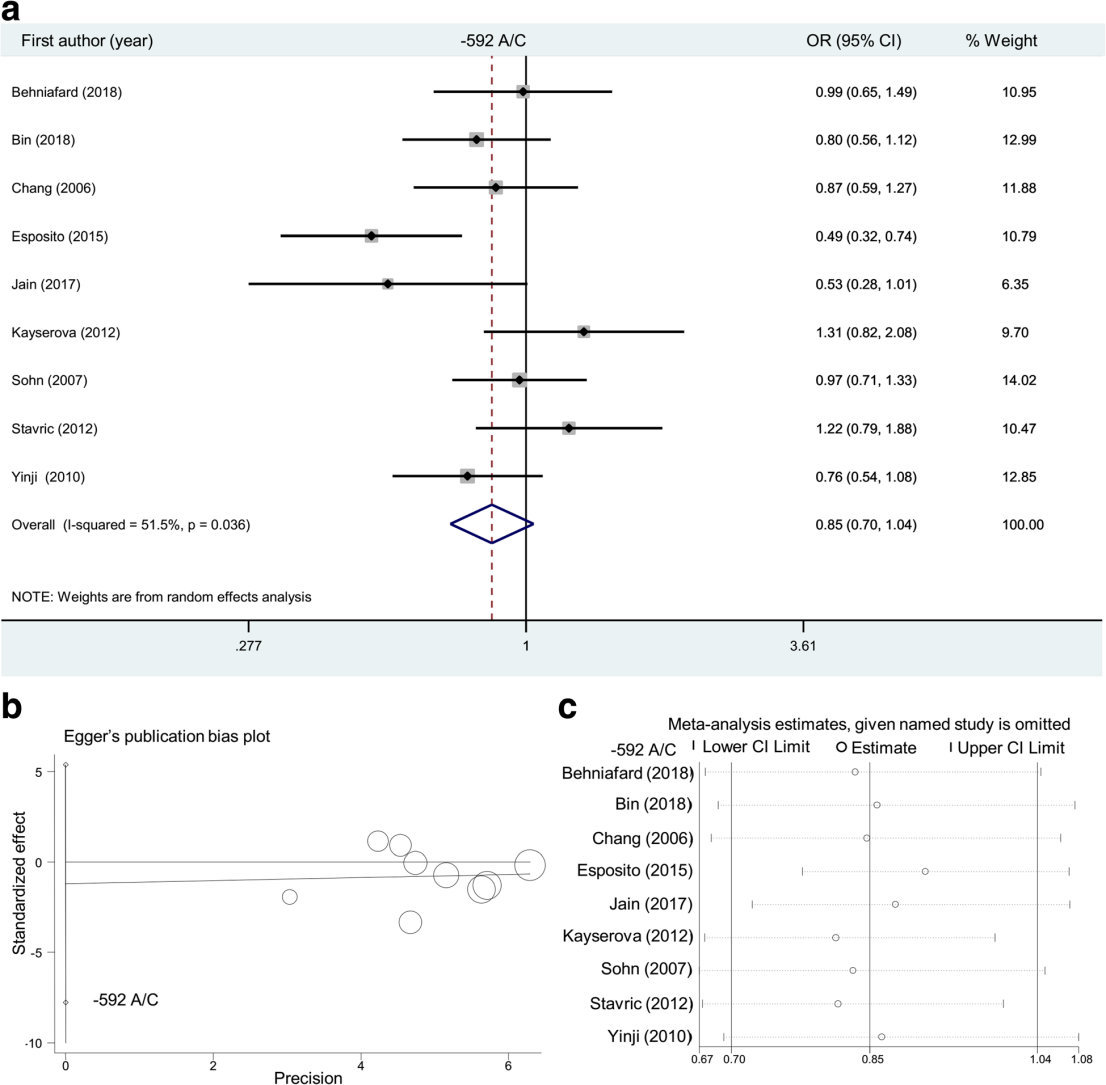
Overall meta-analysis of *IL-10* -592 A/C SNP under the allelic model. (**a**) Forest plot; (**b**) Egger’s publication bias plot; (**c**) Sensitivity analysis

For the *IL-10* -592 A/C SNP (Table 4), nine case-control studies (1032/1363) were enrolled in the allelic model, while eight studies (938/1177) were in enrolled in other models. As shown in Table 4, we only observed positive results in the overall meta-analysis under AC vs. AA (*P* = 0.039, OR = 0.77) and AC + CC vs. AA (*P* = 0.026, OR = 0.76) as well as in the subgroup analysis of “Asian” under allele C vs. A (*P* = 0.033, OR = 0.85) and AC + CC vs. AA (*P* = 0.049, OR = 0.77) but not others (all *P* > 0.05). It is worth mentioning that the negative results were observed in the “Asian/Y” subgroup, under all genetic models, when only the studies with the *P* value of HWE > 0.05 were included in the analysis of “Asian” subgroup. The relative forest plots are displayed in Fig. 4a and Additional file 2: Figures S7-S9. The above outcomes did not provide evidence of the strong association between *IL-10* -592 A/C SNP and atopic dermatitis susceptibility.

**Table 4:** Meta-analysis of *IL-10* -592 A/C SNP

Genetic model	Group	Study number	Case/Control	*P*	OR (95% CI)
Allele C vs. A	Overall	9	1032/1363	0.109	0.85 (0.70~1.04)
	Asian	6	774/844	0.033	0.85 (0.73~0.99)
	Caucasian	3	258/519	0.788	0.92 (0.49~1.73)
	Y	8	994/1325	0.204	0.88 (0.73~1.07)
	Asian/Y	5	736/806	0.086	0.87 (0.74~1.02)
	Caucasian/Y	3	258/519	0.788	0.92 (0.49~1.73)
CC vs. AA	Overall	8	938/1177	0.227	0.72 (0.42~1.23)
	Asian	5	680/658	0.353	0.75 (0.40~1.38)
	Caucasian	3	258/519	0.582	0.71 (0.21~2.38)
	Y	7	900/1139	0.349	0.80 (0.50~1.28)
	Asian/Y	4	642/620	0.471	0.86 (0.56~1.30)
	Caucasian/Y	3	258/519	0.582	0.71 (0.21~2.38)
AC vs. AA	Overall	8	938/1177	0.039	0.77 (0.59~0.99)
	Asian	5	680/658	0.061	0.77 (0.58~1.01)
	Caucasian	3	258/519	0.387	0.76 (0.41~1.41)
	Y	7	900/1139	0.093	0.80 (0.62~1.04)
	Asian/Y	4	642/620	0.146	0.81 (0.61~1.08)
	Caucasian/Y	3	258/519	0.387	0.76 (0.41~1.41)
AC + CC vs. AA	Overall	8	938/1177	0.026	0.76 (0.60~0.97)
	Asian	5	680/658	0.049	0.77 (0.59~1.00)
	Caucasian	3	258/519	0.290	0.73 (0.41~1.31)
	Y	7	900/1139	0.069	0.80 (0.62~1.02)
	Asian/Y	4	642/620	0.128	0.81 (0.62~1.06)
	Caucasian/Y	3	258/519	0.290	0.73 (0.41~1.31)
CC vs. AA + AC	Overall	8	938/1177	0.416	0.86 (0.61~1.23)
	Asian	5	680/658	0.194	0.83 (0.62~1.10)
	Caucasian	3	258/519	0.892	0.94 (0.41~2.19)
	Y	7	900/1139	0.597	0.91 (0.63~1.30)
	Asian/Y	4	642/620	0.324	0.86 (0.64~1.16)
	Caucasian/Y	3	258/519	0.892	0.94 (0.41~2.19)
Carrier C vs. A	Overall	8	938/1177	0.170	0.90 (0.77~1.05)
	Asian	5	680/658	0.180	0.88 (0.73~1.06)
	Caucasian	3	258/519	0.640	0.94 (0.71~1.24)
	Y	7	900/1139	0.244	0.91 (0.77~1.07)
	Asian/Y	4	642/620	0.273	0.90 (0.74~1.09)
	Caucasian/Y	3	258/519	0.640	0.94 (0.71~1.24)

*OR* odds ratio, *Y P* value of HWE >0.05, *CI* confidence interval

### Heterogeneity, publication bias and sensitivity analysis

As shown in Additional file 1: Table S2, a random-effect model (DerSimonian and Laird method) was used under the heterozygotic and dominant models in the meta-analysis of the *IL-10* -1082 A/G SNP; allelic, homozygotic, heterozygotic, dominant and recessive models of the − 819 T/C SNP; and allelic, homozygotic, recessive models of the − 592 A/C SNP (all I^2^ > 50% or *P* < 0.05). In addition, we did not observe a large publication bias in most above analyses (Additional file 1: Table S3, *P* > 0.05), only apart from the Egger’s test under the allelic (*P* = 0.028) and heterozygotic (*P* = 0.021) models of the − 819 T/C SNP. Egger’s publication bias plots are displayed in Figs. 2b, 3b and 4b. Our sensitivity analyses in Figs. 2c, 3c and 4c also suggested the statistical stability of pooling outcomes under the allelic models. Similar data were observed in other models (data not shown).

## Discussion

In 2013, Chen, et al. included seven case-control studies [17–19, 21, 32, 34, 35] to perform a meta-analysis of the association between the *IL-10* -1082 A/G polymorphism and the risk of atopic dermatitis [23]. In the present study, we collected the available data from diverse sources and finally added another eight new case-control studies [15, 16, 20, 30, 31, 33, 36, 37] to conduct another updated meta-analysis under the genetic models of allele A vs. G, GG vs. AA, AG vs AA, AG + GG vs AA, GG vs AA+AG and carrier A vs. G. Subgroup analyses stratified by ethnicity, country and HWE were also conducted. Our data failed to support the strong genetic link between the *IL-10* -1082 A/G SNP and the risk of atopic dermatitis, which is in line with the outcomes of the above meta-analyses. Recently, another meta-analysis by Zhao, et al. [39] reported the same result on the potential association between *IL-10* -1082 A/G SNP and atopic dermatitis susceptibility under the recessive model in the Association population. However, only three case-control studies [20, 30, 37] were enrolled in this comparison, and *P* value of HWE < 0.05 in controls of two studies [20, 37] were detected.

With regards to *IL-10* -819 T/C SNP, a meta-analysis containing nine case-control studies was performed under the six genetic models. The subgroup analysis by the ethnicity was also performed, when the studies with the *P* value of HWE < 0.05 were removed. We found that *IL-10* -819 T/C SNP may not be strongly linked to the risk of atopic dermatitis. Even though the potential positive conclusion was observed under the recessive model in the Caucasian populations [39], only two case-control studies [17, 35] were enrolled for synthetic analysis. The linkage disequilibrium between the *IL-10* -819 T/C and − 592 C/A polymorphisms exists in some reports [15, 21]. However, different genotype frequencies were also reported in other reports [17, 18, 35, 37]. Here, we also pooled the available data to perform a meta-analysis, which did not strongly support the genetic relationship between the *IL-10* -592 C/A SNP and the risk of atopic dermatitis. This is also in line with the conclusion of Zhao, et al. [39].

Our meta-analysis exhibits several advantages. First, all eligible studies contain population-based negative control subjects. Second, our results of Begg’s and Egger’s tests ruled out the presence of large publication bias. Third, our sensitivity analyses support the stability of pooling results.

Despite this, some disadvantages still may limit our statistical evaluation. First, as in other meta-analyses, a small sample size was enrolled in some analyses. For example, less than ten case-control studies were included in the overall meta-analysis of the *IL-10* -819 T/C and − 592 C/A polymorphisms and all the stratification analyses. For the *IL-10* -819 T/C SNP, only two case-control studies [17, 35] were included in the subgroup analysis of “Caucasian”. Only three case-control studies were obtained in the subgroup analysis of “Poland” for *IL-10* -1082 A/G polymorphism, and less than three studies were for the other subgroup analyses of “Country”, such as “China” or “India”. Second, a high heterogeneity level among studies was observed in some overall meta-analyses. Third, the genotypic distribution of control groups in some studies was not in line with the Hardy-Weinberg equilibrium. Fourth, of the eligible case-control studies, only allelic frequency data were extracted in one study [21], and the combined genotypic frequency (AG + GG) of the *IL-10* -1082 A/G polymorphism was obtained in another study [31]. Fifth, even though we obtained haplotypic data for the − 1082 A/G, − 819 T/C, − 592 A/C polymorphisms within the *IL-10* genes, including “ACC”, “ATA”, and “GCC”, very limited data resulted in the failure of related meta-analysis. Sixth, one meta-analysis reported that the − 590 C/T polymorphism within the *IL-4 (interleukin-4)* gene may be linked to the risk of atopic dermatitis, especially for Asian children [40]. Considering the extensive role of interleukins (ILs) in the immune responses of diverse organisms [41–45], it is important to analyse the combined effects of different interleukin gene variants in either the resistance or susceptibility to genetic disorders, including many skin disorders, for a more objective and comprehensive assessment. However, we focused on the role of three SNPs of the *IL-10* gene, one member in the interleukin gene family, in the susceptibility to atopic dermatitis in this study, based on the limitations of study publications. Given the contextual effects of the polymorphism and the diversity of the heterozygotic response, more interleukin gene variant-associated association investigations by additional adjusted clinical or environmental factors should be conducted when we can obtain access to more association investigation data for years to come.

## Conclusion

Overall, based on the current case-control association data, *IL-10* -1082 A/G, − 819 T/C and − 592 A/C polymorphisms may not be strongly related to atopic dermatitis susceptibility, which would be greatly strengthened by a larger sample size.

## Additional files

Additional file 1: **Table S1.** NOS assessment system. **Table S2.** Heterogeneity evaluation. **Table S3.** Publication bias assessment. (DOCX 43 kb)

Additional file 2: **Figure S1.** Subgroup analysis of the *IL-10* -1082 A/G polymorphism according to ethnicity under the allele G vs. A model. **Figure S2.** Subgroup analysis of the *IL-10* -1082 A/G polymorphism according to ethnicity under the AG vs. AA model. **Figure S3.** Subgroup analysis of the *IL-10* -1082 A/G polymorphism according to ethnicity under the AG + GG vs. AA model. **Figure S4.** Subgroup analysis of the *IL-10* -819 T/C polymorphism according to ethnicity under the allele C vs. T model. **Figure S5.** Subgroup analysis of the *IL-10* -819 T/C polymorphism according to ethnicity under the TC vs. TT model. **Figure S6.** Subgroup analysis of the *IL-10* -819 T/C polymorphism according to ethnicity under the TC + CC vs. TT model. **Figure S7.** Subgroup analysis of *IL-10* -592 A/C polymorphism according to ethnicity under the allele C vs. A model. **Figure S8.** Subgroup analysis of *IL-10* -592 A/C polymorphism according to ethnicity under the AC vs. AA model. **Figure S9.** Subgroup analysis of *IL-10* -592 A/C polymorphism according to ethnicity under the AC + CC vs. AA model. (ZIP 2797 kb)

## Abbreviations

ARMS: amplification refractory mutational system
ASPCR: allele-specific PCR
CI: confidence interval
CNKI: China National Knowledge Infrastructure
FLG: filaggrin
GWAS: genome-wide association study
HWE: Hardy-Weinberg equilibrium
IL-10: Interleukin-10
KASP: Kompetitive Allele Specific PCR
NOS: Newcastle-Ottawa quality assessment Scale
OR: odds ratio
PB: population-based control
PCR: polymerase chain reaction
PRISMA: systematic reviews and meta-analyses
RFLP: restriction fragment-length polymorphism
SSP: sequence specific primer
WOS: Web of Science
