# Supplementary material for: Genetic relationship between IL-10 gene polymorphisms and the risk of clinical atopic dermatitis
Source: BMC Med Genet. 2019 May 17;20:83. doi: 10.1186/s12881-019-0817-8 (PMC6525399; doi:10.1186/s12881-019-0817-8)
Supplement: Supplementary file 1 — Table S1. NOS assessment system. Table S2. Heterogeneity evaluation. Table S3. Publication bias assessment. (DOCX 43 kb) [file 12881_2019_817_MOESM1_ESM.docx]

Table S1 NOS assessment system.

| **First author** | **Year** | **Case Definition adequate?** | **Representativeness of cases** | **Selection of controls** | **Definition of controls** | **Important factors of comparability** | **Other factors of comparability** | **Secure record of exposure** | **Same method of ascertainment** | **Non-response rate** | **NOS score** |
| --- | --- | --- | --- | --- | --- | --- | --- | --- | --- | --- | --- |
| **Arkwright** | **2001** | *1* | *1* | *1* | *1* | *1* | *1* | *0* | *1* | *1* | **8** |
| **Babic** | **2016** | *1* | *1* | *1* | *1* | *0* | *0* | *1* | *1* | *0* | **6** |
| **Behniafard** | **2018** | *1* | *1* | *1* | *1* | *0* | *0* | *1* | *1* | *1* | **7** |
| **Bin** | **2018** | *1* | *1* | *1* | *1* | *1* | *0* | *1* | *1* | *1* | **8** |
| **Chang** | **2006** | *1* | *1* | *1* | *1* | *1* | *1* | *0* | *1* | *1* | **8** |
| **Esposito** | **2015** | *1* | *1* | *1* | *1* | *1* | *1* | *1* | *1* | *0* | **8** |
| **Jain** | **2017** | *1* | *1* | *1* | *1* | *1* | *0* | *1* | *1* | *1* | **8** |
| **Kayserova** | **2012** | *1* | *1* | *1* | *1* | *1* | *1* | *0* | *1* | *0* | **7** |
| **Lesiak** | **2011** | *1* | *1* | *1* | *1* | *1* | *0* | *0* | *1* | *1* | **7** |
| **Lesiak** | **2014** | *1* | *1* | *1* | *1* | *1* | *0* | *0* | *1* | *1* | **7** |
| **Reich** | **2003** | *1* | *1* | *1* | *1* | *1* | *1* | *0* | *1* | *1* | **8** |
| **Sheng** | **2018** | *1* | *1* | *1* | *1* | *1* | *1* | *0* | *1* | *0* | **7** |
| **Sohn** | **2007** | *1* | *1* | *1* | *1* | *0* | *0* | *0* | *1* | *1* | **6** |
| **Stavric** | **2012** | *1* | *1* | *1* | *1* | *1* | *1* | *0* | *1* | *0* | **7** |
| **Yinji** | **2010** | *1* | *1* | *1* | *1* | *1* | *0* | *0* | *1* | *0* | **6** |
| **Zakrzewski** | **2010** | *1* | *1* | *1* | *1* | *0* | *0* | *0* | *1* | *1* | **6** |

*NOS* Newcastle-Ottawa quality assessment Scale

Table S2. Heterogeneity evaluation

| **SNPs** | **allele** | | | **homozygote** | | | **heterozygote** | | | **dominant** | | | **recessive** | | | **carrier** | | |
| --- | --- | --- | --- | --- | --- | --- | --- | --- | --- | --- | --- | --- | --- | --- | --- | --- | --- | --- |
|  | **I^2^** | ***P*** | **M** | **I^2^** | ***P*** | **M** | **I^2^** | ***P*** | **M** | **I^2^** | ***P*** | **M** | **I^2^** | ***P*** | **M** | **I^2^** | ***P*** | **M** |
| **-1082A/G** | 27.4% | 0.161 | f | 5.0% | 0.395 | f | 66.9% | <0.05 | r | 59.9% | 0.002 | r | 45.4% | 0.050 | f | 0.0% | 0.834 | f |
| **-819T/C** | 71.4% | <0.001 | r | 71.7% | 0.001 | r | 70.3% | 0.001 | r | 71.4% | <0.001 | r | 64.5% | 0.006 | r | 33.4% | 0.162 | f |
| **-592A/C** | 51.5% | 0.036 | r | 52.2% | 0.041 | r | 29.7% | 0.191 | f | 35.2% | 0.147 | f | 60.8% | 0.013 | r | 0.0% | 0.430 | f |

*SNPs* single nucleotide polymorphisms, *M* Fixed or random models used in association test, *f* fixed, *r* random

Table S3. Publication bias assessment

| **SNPs** | **allele** | | **homozygote** | | **heterozygote** | | **dominant** | | **recessive** | | **carrier** | |
| --- | --- | --- | --- | --- | --- | --- | --- | --- | --- | --- | --- | --- |
|  | *P*-Begg’s test | *P*-Egger’s test | *P*-Begg’s test | *P*-Egger’s test | *P*-Begg’s test | *P*-Egger’s test | *P*-Begg’s test | *P*-Egger’s test | *P*-Begg’s test | *P*-Egger’s test | *P*-Begg’s test | *P*-Egger’s test |
| **-1082A/G** | 0.784 | 0.352 | 1.000 | 0.795 | 0.807 | 0.469 | 0.622 | 0.438 | 0.938 | 0.807 | 0.714 | 0.287 |
| **-819T/C** | 0.175 | 0.028 | 0.386 | 0.021 | 0.266 | 0.150 | 0.536 | 0.119 | 0.536 | 0.306 | 0.386 | 0.105 |
| **-592A/C** | 0.917 | 0.681 | 0.902 | 0.396 | 0.536 | 0.717 | 0.711 | 0.597 | 1.000 | 0.792 | 1.000 | 0.906 |

*SNPs* single nucleotide polymorphisms
