# Supplementary figures and images for: Genetic relationship between IL-10 gene polymorphisms and the risk of clinical atopic dermatitis
Source: BMC Med Genet. 2019 May 17;20:83. doi: 10.1186/s12881-019-0817-8 (PMC6525399; doi:10.1186/s12881-019-0817-8)

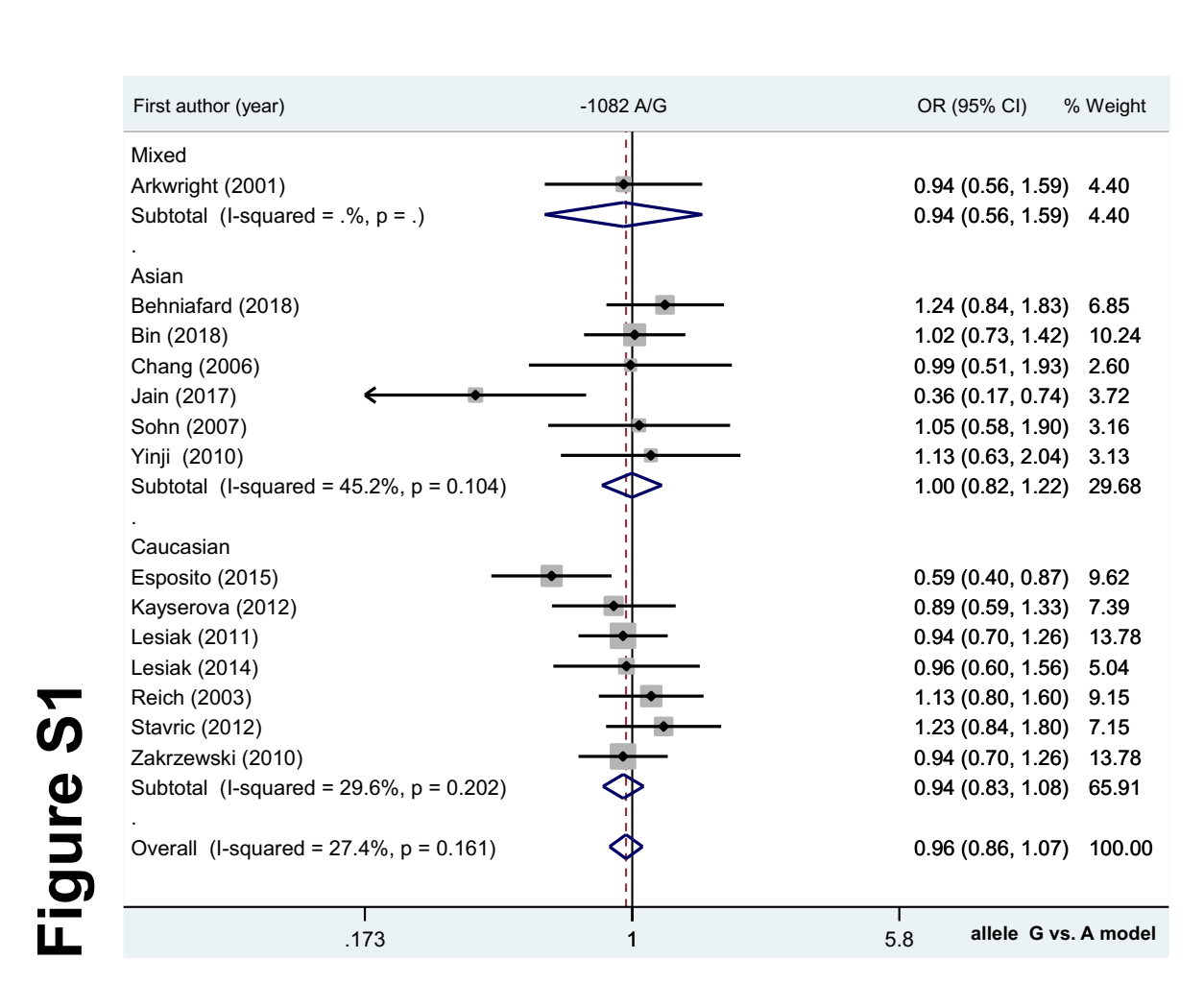

Supplement: Supplementary file 2 — Figure S1. Subgroup analysis of the IL-10 -1082 A/G polymorphism according to ethnicity under the allele G vs. A model. Figure S2. Subgroup analysis of the IL-10 -1082 A/G polymorphism according to ethnicity under the AG vs. AA model. Figure S3. Subgroup analysis of the IL-10 -1082 A/G polymorphism according to ethnicity under the AG + GG vs. AA model. Figure S4. Subgroup analysis of the IL-10 -819 T/C polymorphism according to ethnicity under the allele C vs. T model. Figure S5. Subgroup analysis of the IL-10 -819 T/C polymorphism according to ethnicity under the TC vs. TT model. Figure S6. Subgroup analysis of the IL-10 -819 T/C polymorphism according to ethnicity under the TC + CC vs. TT model. Figure S7. Subgroup analysis of IL-10 -592 A/C polymorphism according to ethnicity under the allele C vs. A model. Figure S8. Subgroup analysis of IL-10 -592 A/C polymorphism according to ethnicity under the AC vs. AA model. Figure S9. Subgroup analysis of IL-10 -592 A/C polymorphism according to ethnicity under the AC + CC vs. AA model. (ZIP 2797 kb) [file 12881_2019_817_MOESM2_ESM.zip › Figure S1R4.tif]

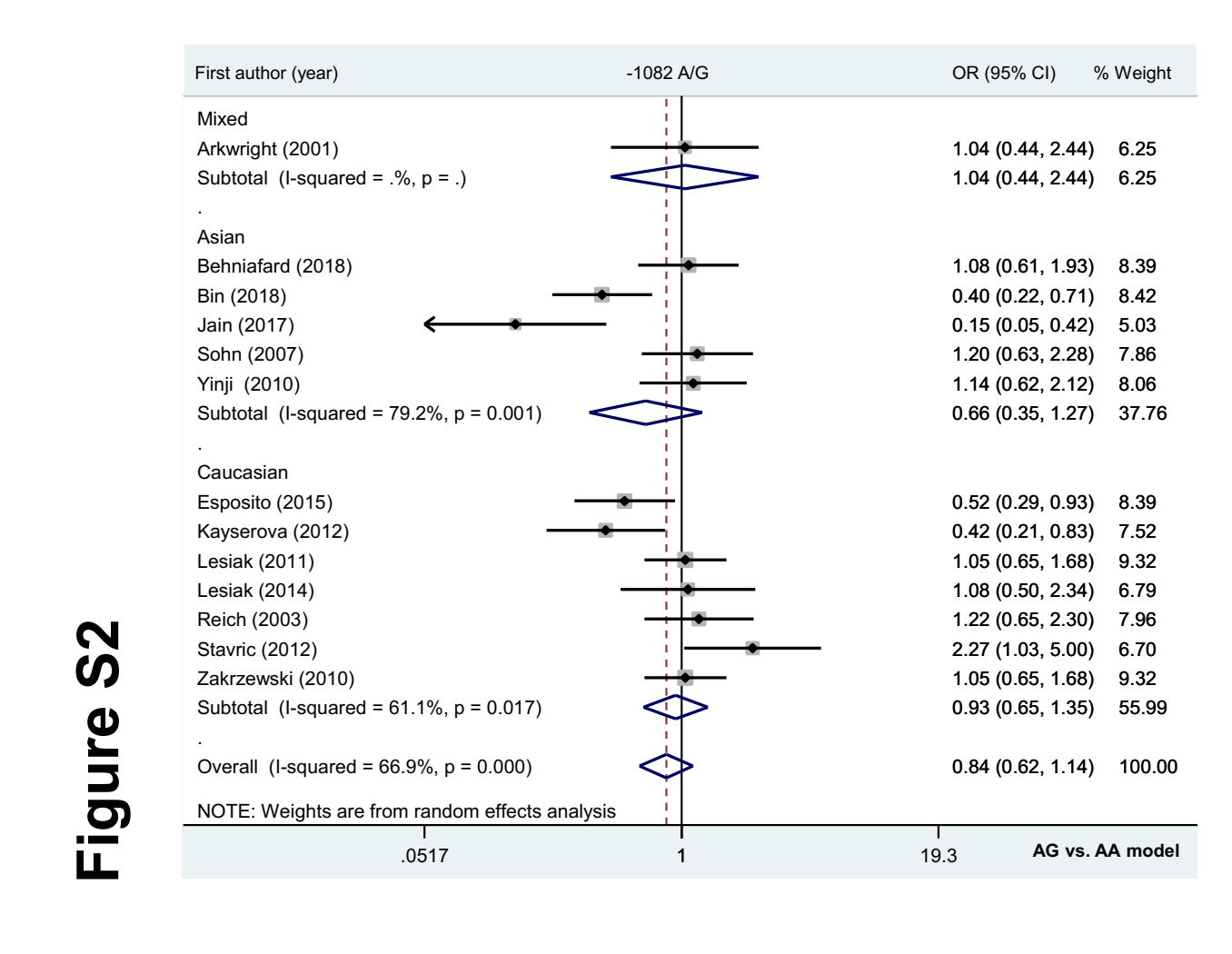

Supplement: Supplementary file 2 — Figure S1. Subgroup analysis of the IL-10 -1082 A/G polymorphism according to ethnicity under the allele G vs. A model. Figure S2. Subgroup analysis of the IL-10 -1082 A/G polymorphism according to ethnicity under the AG vs. AA model. Figure S3. Subgroup analysis of the IL-10 -1082 A/G polymorphism according to ethnicity under the AG + GG vs. AA model. Figure S4. Subgroup analysis of the IL-10 -819 T/C polymorphism according to ethnicity under the allele C vs. T model. Figure S5. Subgroup analysis of the IL-10 -819 T/C polymorphism according to ethnicity under the TC vs. TT model. Figure S6. Subgroup analysis of the IL-10 -819 T/C polymorphism according to ethnicity under the TC + CC vs. TT model. Figure S7. Subgroup analysis of IL-10 -592 A/C polymorphism according to ethnicity under the allele C vs. A model. Figure S8. Subgroup analysis of IL-10 -592 A/C polymorphism according to ethnicity under the AC vs. AA model. Figure S9. Subgroup analysis of IL-10 -592 A/C polymorphism according to ethnicity under the AC + CC vs. AA model. (ZIP 2797 kb) [file 12881_2019_817_MOESM2_ESM.zip › Figure S2R4.tif]

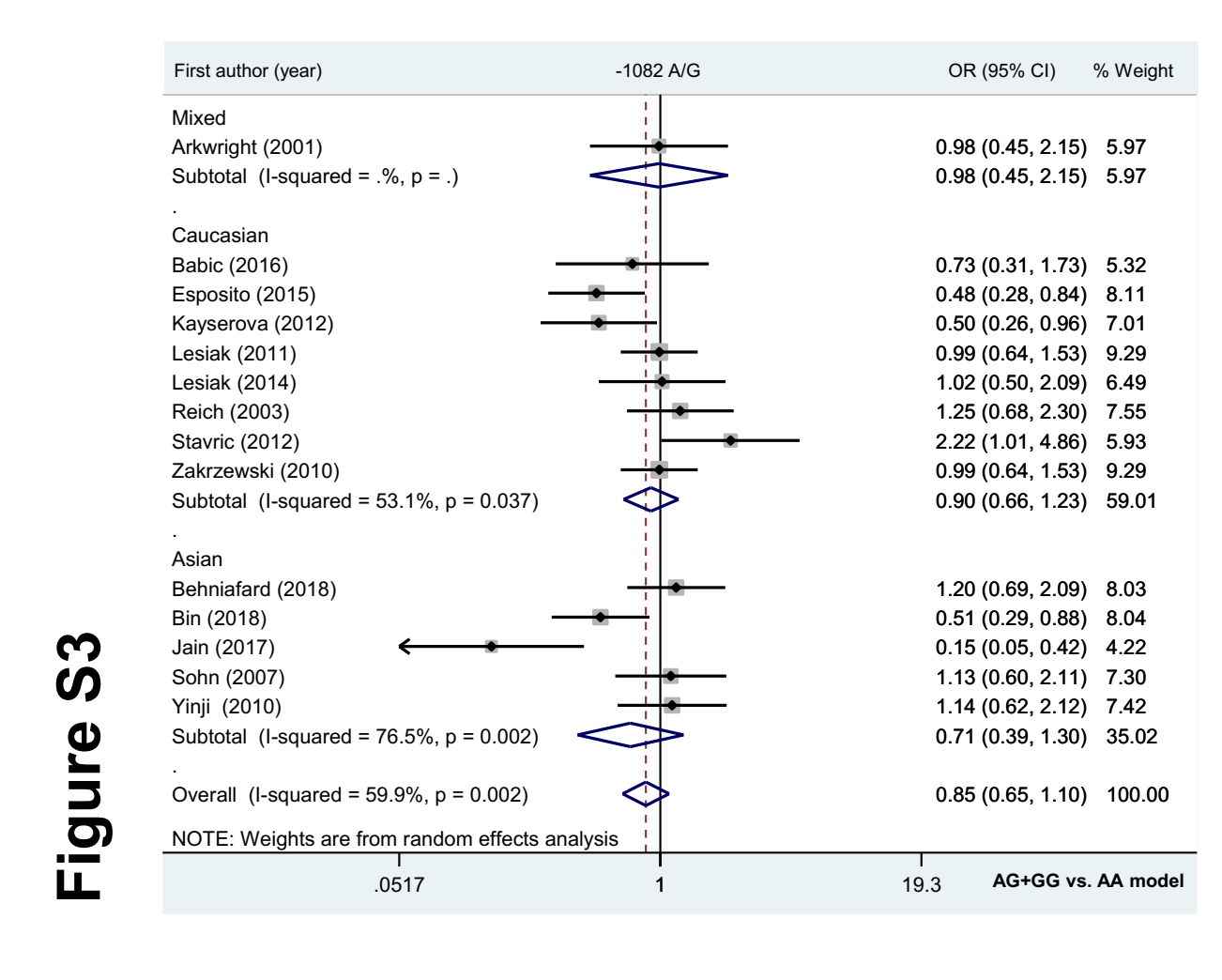

Supplement: Supplementary file 2 — Figure S1. Subgroup analysis of the IL-10 -1082 A/G polymorphism according to ethnicity under the allele G vs. A model. Figure S2. Subgroup analysis of the IL-10 -1082 A/G polymorphism according to ethnicity under the AG vs. AA model. Figure S3. Subgroup analysis of the IL-10 -1082 A/G polymorphism according to ethnicity under the AG + GG vs. AA model. Figure S4. Subgroup analysis of the IL-10 -819 T/C polymorphism according to ethnicity under the allele C vs. T model. Figure S5. Subgroup analysis of the IL-10 -819 T/C polymorphism according to ethnicity under the TC vs. TT model. Figure S6. Subgroup analysis of the IL-10 -819 T/C polymorphism according to ethnicity under the TC + CC vs. TT model. Figure S7. Subgroup analysis of IL-10 -592 A/C polymorphism according to ethnicity under the allele C vs. A model. Figure S8. Subgroup analysis of IL-10 -592 A/C polymorphism according to ethnicity under the AC vs. AA model. Figure S9. Subgroup analysis of IL-10 -592 A/C polymorphism according to ethnicity under the AC + CC vs. AA model. (ZIP 2797 kb) [file 12881_2019_817_MOESM2_ESM.zip › Figure S3R4.tif]

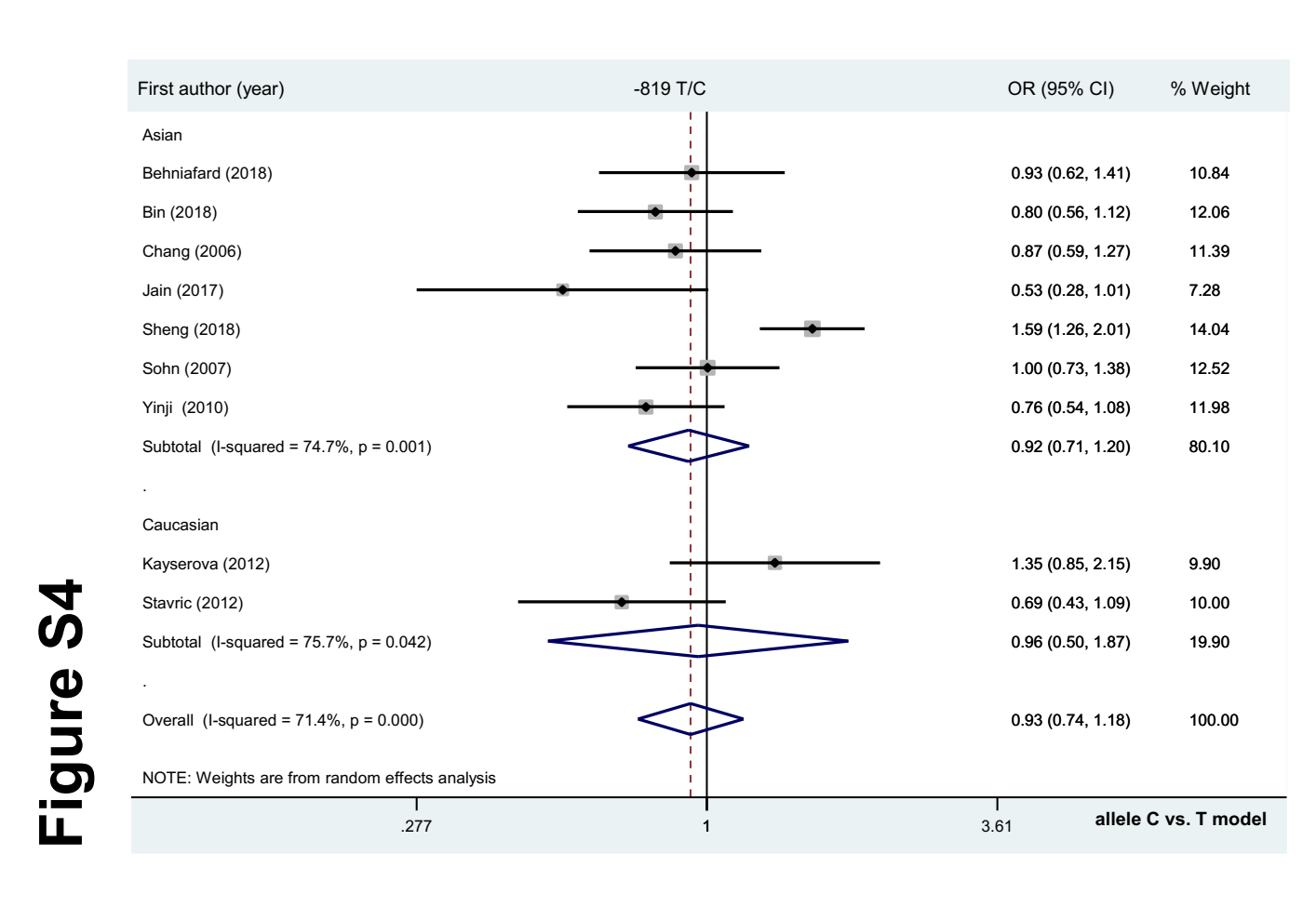

Supplement: Supplementary file 2 — Figure S1. Subgroup analysis of the IL-10 -1082 A/G polymorphism according to ethnicity under the allele G vs. A model. Figure S2. Subgroup analysis of the IL-10 -1082 A/G polymorphism according to ethnicity under the AG vs. AA model. Figure S3. Subgroup analysis of the IL-10 -1082 A/G polymorphism according to ethnicity under the AG + GG vs. AA model. Figure S4. Subgroup analysis of the IL-10 -819 T/C polymorphism according to ethnicity under the allele C vs. T model. Figure S5. Subgroup analysis of the IL-10 -819 T/C polymorphism according to ethnicity under the TC vs. TT model. Figure S6. Subgroup analysis of the IL-10 -819 T/C polymorphism according to ethnicity under the TC + CC vs. TT model. Figure S7. Subgroup analysis of IL-10 -592 A/C polymorphism according to ethnicity under the allele C vs. A model. Figure S8. Subgroup analysis of IL-10 -592 A/C polymorphism according to ethnicity under the AC vs. AA model. Figure S9. Subgroup analysis of IL-10 -592 A/C polymorphism according to ethnicity under the AC + CC vs. AA model. (ZIP 2797 kb) [file 12881_2019_817_MOESM2_ESM.zip › Figure S4R4.tif]

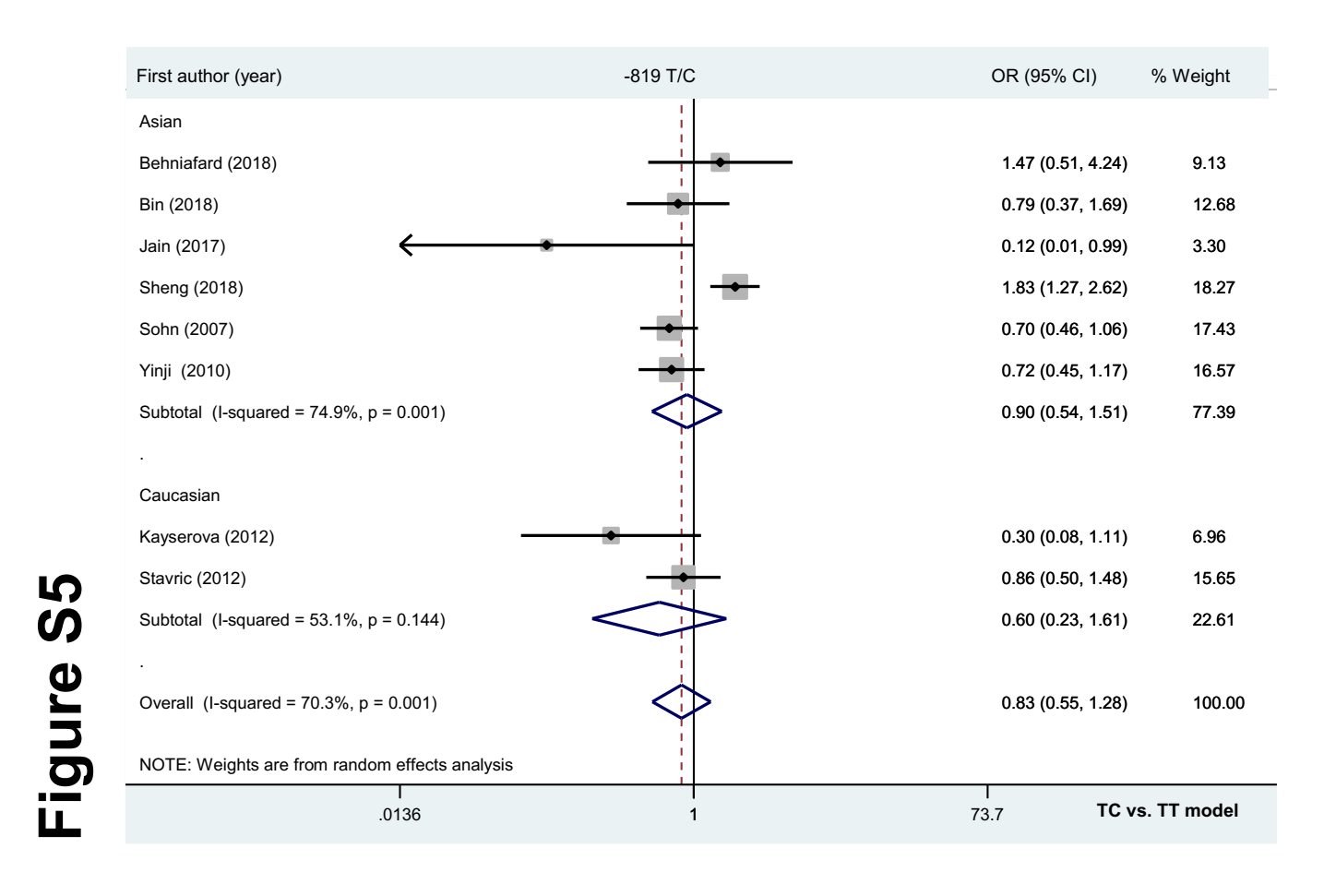

Supplement: Supplementary file 2 — Figure S1. Subgroup analysis of the IL-10 -1082 A/G polymorphism according to ethnicity under the allele G vs. A model. Figure S2. Subgroup analysis of the IL-10 -1082 A/G polymorphism according to ethnicity under the AG vs. AA model. Figure S3. Subgroup analysis of the IL-10 -1082 A/G polymorphism according to ethnicity under the AG + GG vs. AA model. Figure S4. Subgroup analysis of the IL-10 -819 T/C polymorphism according to ethnicity under the allele C vs. T model. Figure S5. Subgroup analysis of the IL-10 -819 T/C polymorphism according to ethnicity under the TC vs. TT model. Figure S6. Subgroup analysis of the IL-10 -819 T/C polymorphism according to ethnicity under the TC + CC vs. TT model. Figure S7. Subgroup analysis of IL-10 -592 A/C polymorphism according to ethnicity under the allele C vs. A model. Figure S8. Subgroup analysis of IL-10 -592 A/C polymorphism according to ethnicity under the AC vs. AA model. Figure S9. Subgroup analysis of IL-10 -592 A/C polymorphism according to ethnicity under the AC + CC vs. AA model. (ZIP 2797 kb) [file 12881_2019_817_MOESM2_ESM.zip › Figure S5R4.tif]

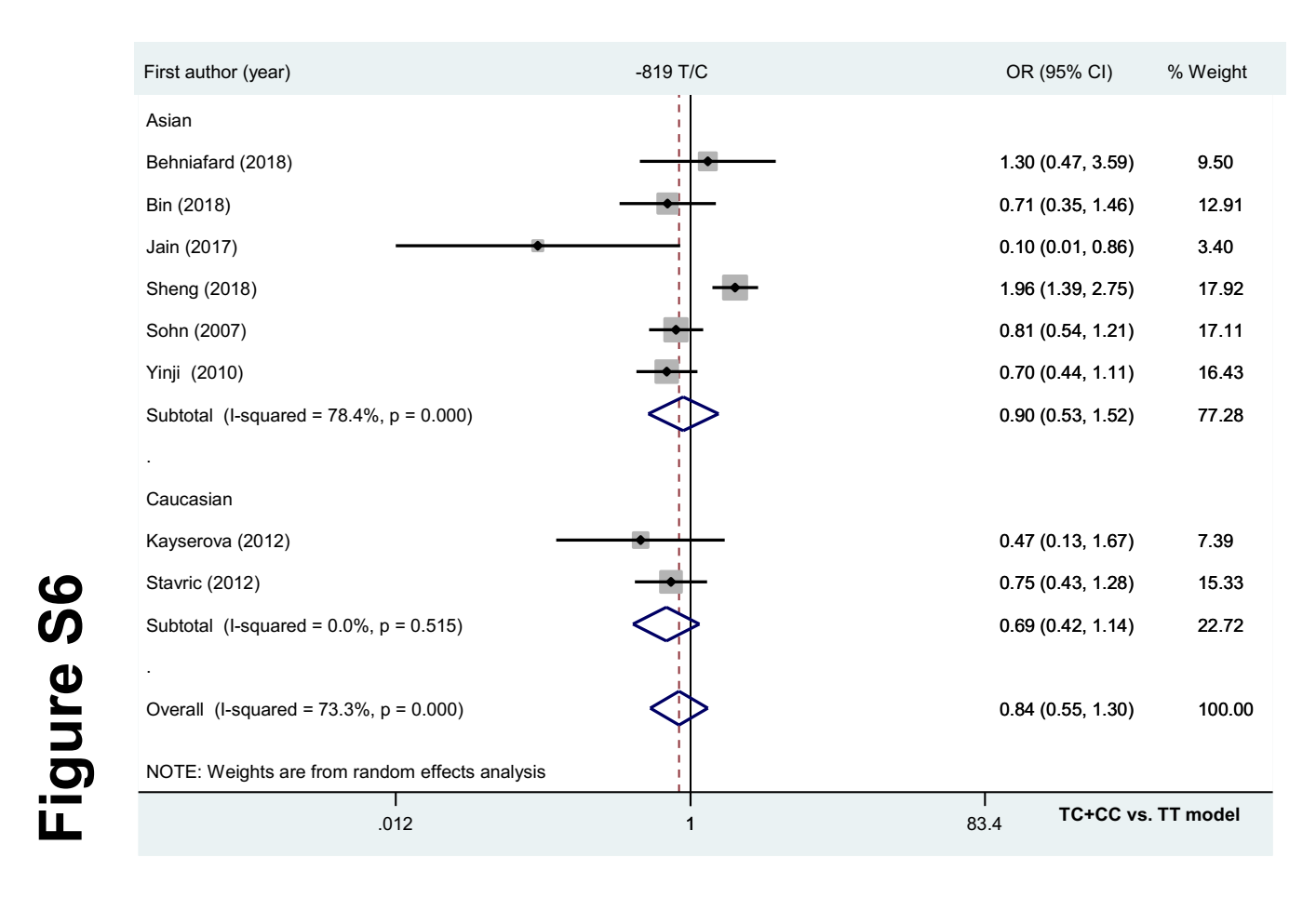

Supplement: Supplementary file 2 — Figure S1. Subgroup analysis of the IL-10 -1082 A/G polymorphism according to ethnicity under the allele G vs. A model. Figure S2. Subgroup analysis of the IL-10 -1082 A/G polymorphism according to ethnicity under the AG vs. AA model. Figure S3. Subgroup analysis of the IL-10 -1082 A/G polymorphism according to ethnicity under the AG + GG vs. AA model. Figure S4. Subgroup analysis of the IL-10 -819 T/C polymorphism according to ethnicity under the allele C vs. T model. Figure S5. Subgroup analysis of the IL-10 -819 T/C polymorphism according to ethnicity under the TC vs. TT model. Figure S6. Subgroup analysis of the IL-10 -819 T/C polymorphism according to ethnicity under the TC + CC vs. TT model. Figure S7. Subgroup analysis of IL-10 -592 A/C polymorphism according to ethnicity under the allele C vs. A model. Figure S8. Subgroup analysis of IL-10 -592 A/C polymorphism according to ethnicity under the AC vs. AA model. Figure S9. Subgroup analysis of IL-10 -592 A/C polymorphism according to ethnicity under the AC + CC vs. AA model. (ZIP 2797 kb) [file 12881_2019_817_MOESM2_ESM.zip › Figure S6R4.tif]

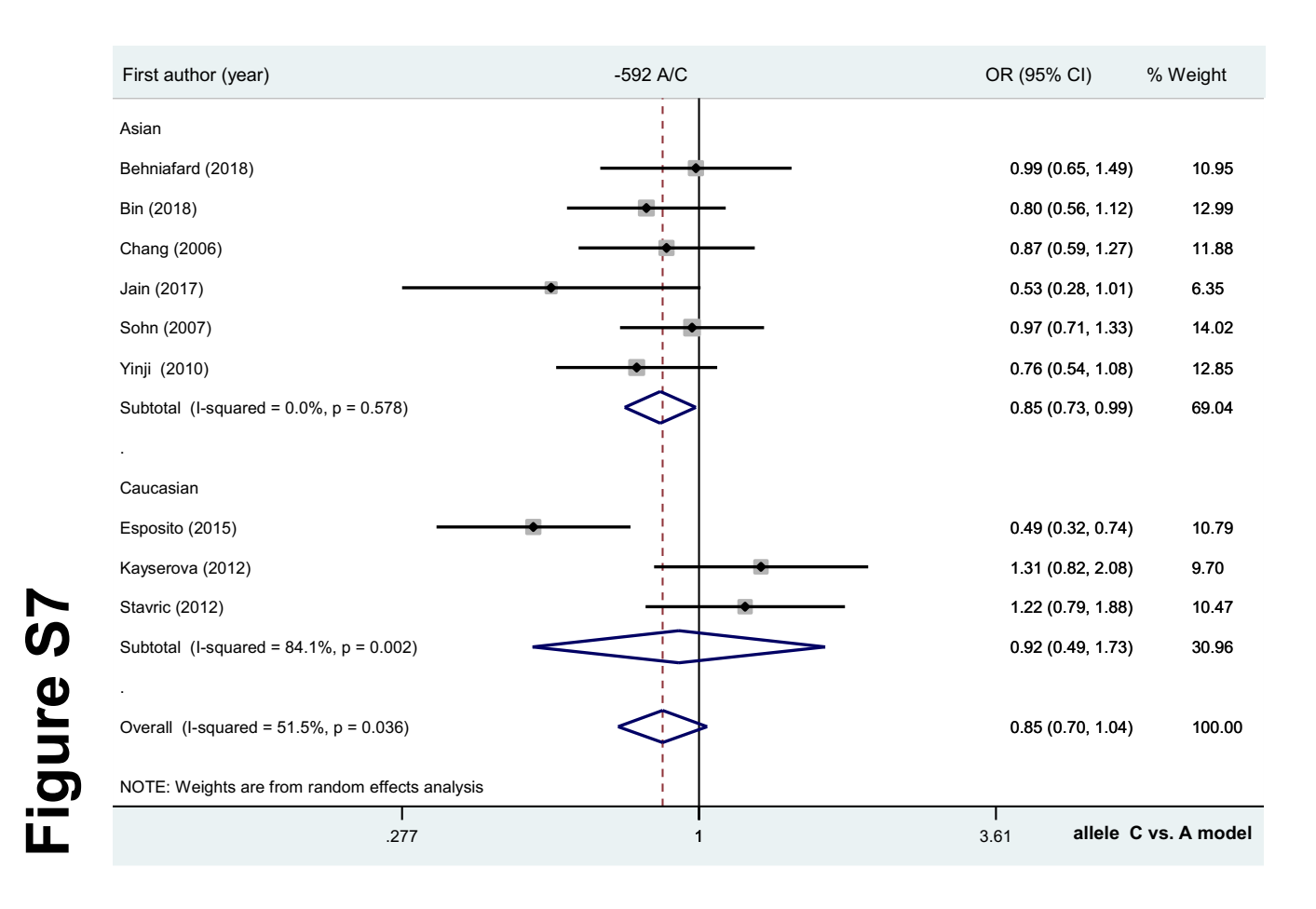

Supplement: Supplementary file 2 — Figure S1. Subgroup analysis of the IL-10 -1082 A/G polymorphism according to ethnicity under the allele G vs. A model. Figure S2. Subgroup analysis of the IL-10 -1082 A/G polymorphism according to ethnicity under the AG vs. AA model. Figure S3. Subgroup analysis of the IL-10 -1082 A/G polymorphism according to ethnicity under the AG + GG vs. AA model. Figure S4. Subgroup analysis of the IL-10 -819 T/C polymorphism according to ethnicity under the allele C vs. T model. Figure S5. Subgroup analysis of the IL-10 -819 T/C polymorphism according to ethnicity under the TC vs. TT model. Figure S6. Subgroup analysis of the IL-10 -819 T/C polymorphism according to ethnicity under the TC + CC vs. TT model. Figure S7. Subgroup analysis of IL-10 -592 A/C polymorphism according to ethnicity under the allele C vs. A model. Figure S8. Subgroup analysis of IL-10 -592 A/C polymorphism according to ethnicity under the AC vs. AA model. Figure S9. Subgroup analysis of IL-10 -592 A/C polymorphism according to ethnicity under the AC + CC vs. AA model. (ZIP 2797 kb) [file 12881_2019_817_MOESM2_ESM.zip › Figure S7R4.tif]

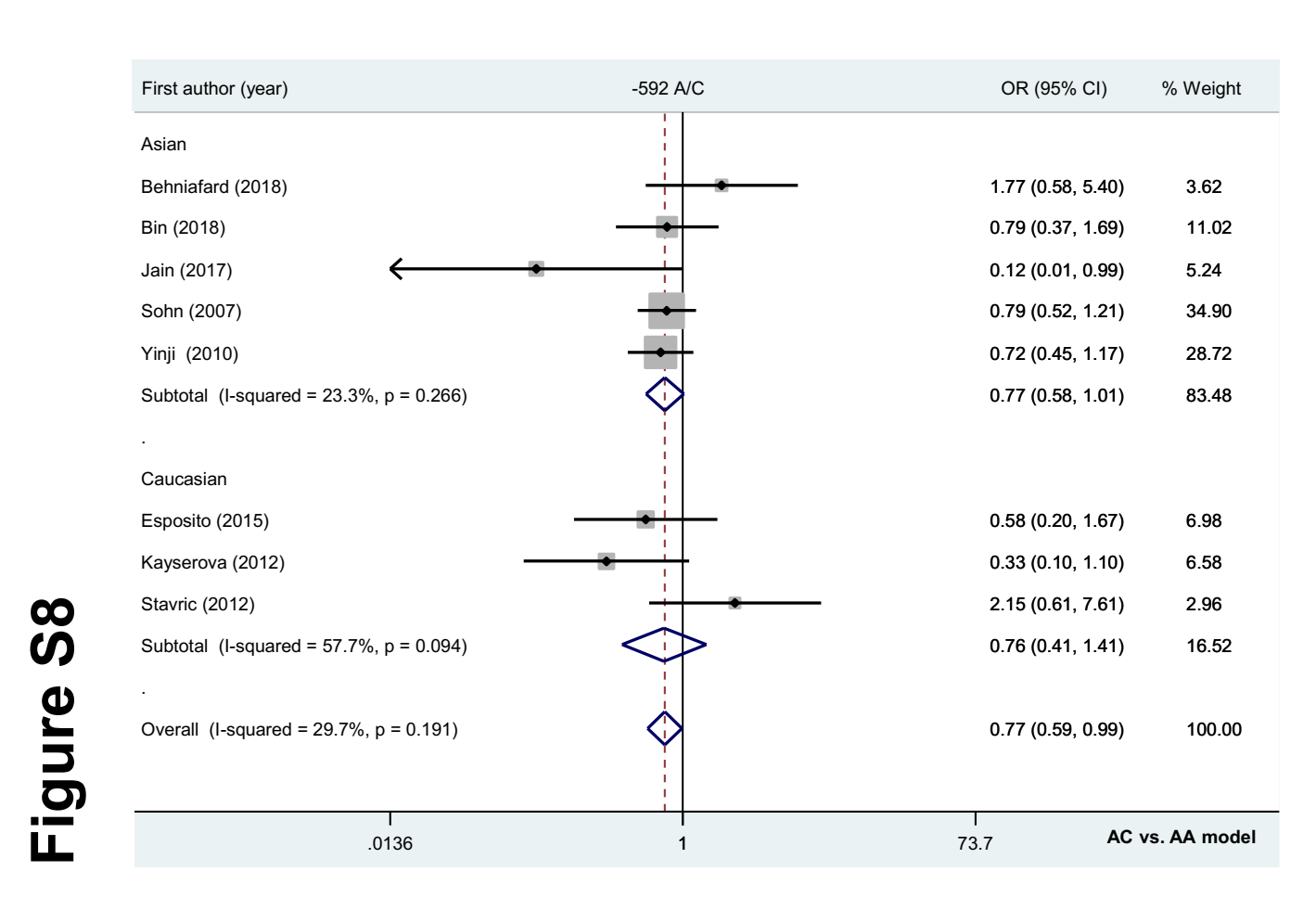

Supplement: Supplementary file 2 — Figure S1. Subgroup analysis of the IL-10 -1082 A/G polymorphism according to ethnicity under the allele G vs. A model. Figure S2. Subgroup analysis of the IL-10 -1082 A/G polymorphism according to ethnicity under the AG vs. AA model. Figure S3. Subgroup analysis of the IL-10 -1082 A/G polymorphism according to ethnicity under the AG + GG vs. AA model. Figure S4. Subgroup analysis of the IL-10 -819 T/C polymorphism according to ethnicity under the allele C vs. T model. Figure S5. Subgroup analysis of the IL-10 -819 T/C polymorphism according to ethnicity under the TC vs. TT model. Figure S6. Subgroup analysis of the IL-10 -819 T/C polymorphism according to ethnicity under the TC + CC vs. TT model. Figure S7. Subgroup analysis of IL-10 -592 A/C polymorphism according to ethnicity under the allele C vs. A model. Figure S8. Subgroup analysis of IL-10 -592 A/C polymorphism according to ethnicity under the AC vs. AA model. Figure S9. Subgroup analysis of IL-10 -592 A/C polymorphism according to ethnicity under the AC + CC vs. AA model. (ZIP 2797 kb) [file 12881_2019_817_MOESM2_ESM.zip › Figure S8R4.tif]

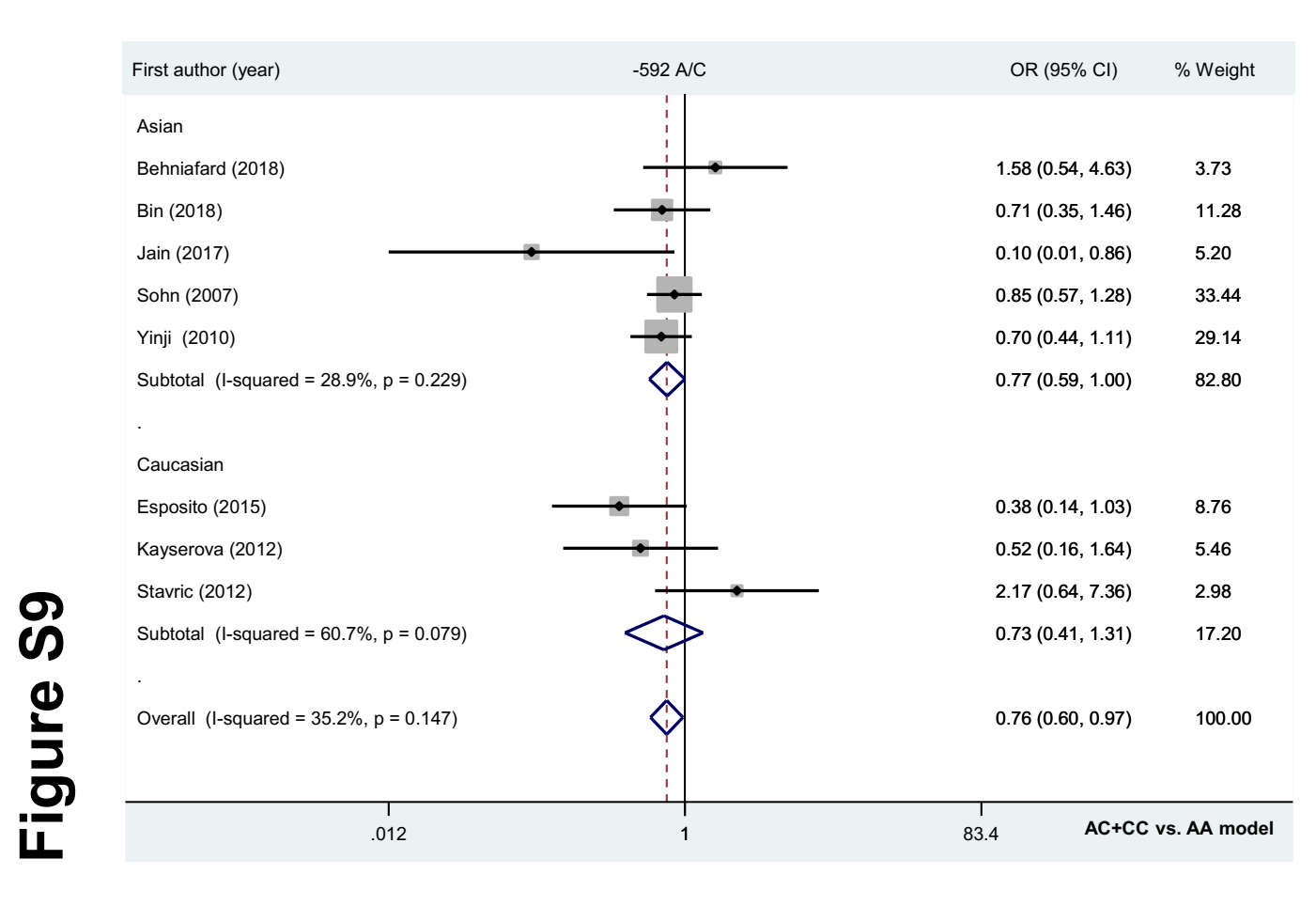

Supplement: Supplementary file 2 — Figure S1. Subgroup analysis of the IL-10 -1082 A/G polymorphism according to ethnicity under the allele G vs. A model. Figure S2. Subgroup analysis of the IL-10 -1082 A/G polymorphism according to ethnicity under the AG vs. AA model. Figure S3. Subgroup analysis of the IL-10 -1082 A/G polymorphism according to ethnicity under the AG + GG vs. AA model. Figure S4. Subgroup analysis of the IL-10 -819 T/C polymorphism according to ethnicity under the allele C vs. T model. Figure S5. Subgroup analysis of the IL-10 -819 T/C polymorphism according to ethnicity under the TC vs. TT model. Figure S6. Subgroup analysis of the IL-10 -819 T/C polymorphism according to ethnicity under the TC + CC vs. TT model. Figure S7. Subgroup analysis of IL-10 -592 A/C polymorphism according to ethnicity under the allele C vs. A model. Figure S8. Subgroup analysis of IL-10 -592 A/C polymorphism according to ethnicity under the AC vs. AA model. Figure S9. Subgroup analysis of IL-10 -592 A/C polymorphism according to ethnicity under the AC + CC vs. AA model. (ZIP 2797 kb) [file 12881_2019_817_MOESM2_ESM.zip › Figure S9R4.tif]
